# Comparative visual outcomes of the first versus second eye following small-incision lenticule extraction (SMILE)

**DOI:** 10.1186/s12886-024-03414-9

**Published:** 2024-04-11

**Authors:** Anzhen Li, Xiaowei Yang, Wei Wang, Wenbin Huang, Hui Ding, Ke Nie, Tan Zhong, Shisi Hu, Zhenduo Yang, Xingwu Zhong

**Affiliations:** 1 State Key Laboratory of Ophthalmology, Zhongshan Ophthalmic Center, Sun Yat-Sen University, Guangdong Provincial Key Laboratory of Ophthalmology Visual Science, Guangdong Provincial Clinical Research Center for Ocular Diseases, Guangzhou, 510060 China; 2 Hainan Eye Hospital and Key Laboratory of Ophthalmology, Zhongshan Ophthalmic Center, Sun Yat-Sen University, Haikou, China

**Keywords:** SMILE, Surgical sequence, Lenticule decentration, High-order-aberration, Modulation transfer function

## Abstract

**Background:**

This study aimed to compare the visual outcomes of the first operated eyes with those of the second operated eyes following small-incision lenticule extraction (SMILE).

**Methods:**

A total of 202 patients (404 eyes) underwent SMILE using the tear film mark centration method for myopia and myopic astigmatism correction. Baseline characteristics, objective optical quality, decentered displacement, induced corneal aberrations, and modulation transfer function (MTF) values were assessed. Linear regression analyzed the relationship between decentration and visual quality parameters, including corneal aberrations and MTF values.

**Results:**

No significant difference was observed in objective visual quality, efficacy, and safety indexes between the two groups (all *P* > 0.05). The average decentered displacement for the first and second surgical eyes was 0.278 ± 0.17 mm and 0.315 ± 0.15 mm, respectively (*P* = 0.002). The horizontal coma in the first surgical eyes were notably lower than in the second (*P* = 0.000). MTF values at spatial frequencies of 5, 10, 15, and 20 cycles/degree (c/d) were higher in the first surgical eyes compared to the second (all *P* < 0.05). Linear regression indicated that high-order aberrations (HOAs), root mean square (RMS) coma, spherical aberration, horizontal coma, vertical coma, and eccentric displacement were all linearly correlated. Furthermore, MTF values exhibited a linear relationship with eccentric displacement across these spatial frequencies.

**Conclusions:**

There was no discernible difference in visual acuity, efficacy, or safety between the two operated eyes. Nonetheless, the first operated eyes exhibited reduced decentered displacement and demonstrated superior outcomes in terms of horizontal coma and MTF values compared to the second operated eyes following SMILE. The variations in visual quality parameters were linearly correlated with decentered displacement.

## Background

Small-incision lenticule extraction (SMILE) has emerged as a preferred technique for correcting myopia and myopic astigmatism due to its superior efficacy, safety, stability, and predictability compared to photorefractive keratectomy, laser in situ keratomileusis (LASIK), and other methods [1–3]. One notable benefit of SMILE is its significant reduction in flap-related complications and diminished postoperative discomfort [4].

Clinically, SMILE surgeries on both eyes tend to be sequential, with both eyes often displaying symmetric functionality and similar anatomical features [5]. While the operational procedures are largely consistent, differences have been observed between the outcomes of the first and second operated eyes. For instance, in cataract surgeries, the first eye operation typically involves less pain and better patient cooperation, and the perceived duration seems notably shorter [6]. In LASIK procedures, patients frequently report increased discomfort during the second eye's operation and show elevated blood pressure levels [7]. Additionally, disparities in epithelium and Bowman’s layer microdistortions post-SMILE between the eyes influence wavefront aberrations, suggesting potential variations in clinical outcomes between the two operated eyes [8].

This study aimed to discern potential discrepancies between the first and second operated eyes post-SMILE by examining visual acuity, safety, efficacy, and visual quality metrics, including corneal wavefront aberrations and MTF values. Furthermore, the underlying reasons for any observed differences between the eyes were explored.

## Methods

This comparative, observational study evaluated 202 patients (404 eyes) who underwent SMILE for the correction of myopia and myopic astigmatism from March 2017 to June 2021 at Hainan Eye Hospital. The research adhered to the tenets of the Declaration of Helsinki and received approval from the Ethics Review Board of Hainan Eye Hospital at the Zhongshan Ophthalmic Center.

Inclusion criteria encompassed patients with stable refraction for a minimum of 2 years preceding the surgery; spherical myopia up to -10.00 diopters (D) and/or myopic astigmatism up to -4.50 D cylinder; age between 18 and 45 years; and accessible preoperative and postoperative records. Exclusion criteria comprised diagnosis of other ocular disorders or pertinent systemic diseases. 

Comprehensive ophthalmic evaluations were conducted preoperatively and three months postoperatively. These included measurements of uncorrected distance visual acuity (UDVA), corrected distance visual acuity (CDVA), and manifest refraction, as well as slit-lamp anterior segment and fundus evaluations. The corrected intraocular pressure (IOP) was determined using a Corvis ST instrument (Oculus, Germany), while the central corneal thickness (CCT) and keratometry were gauged using a Scheimpflug system (Pentacam HR, Oculus, Germany).

### Surgical procedure

**Fig. 1:**
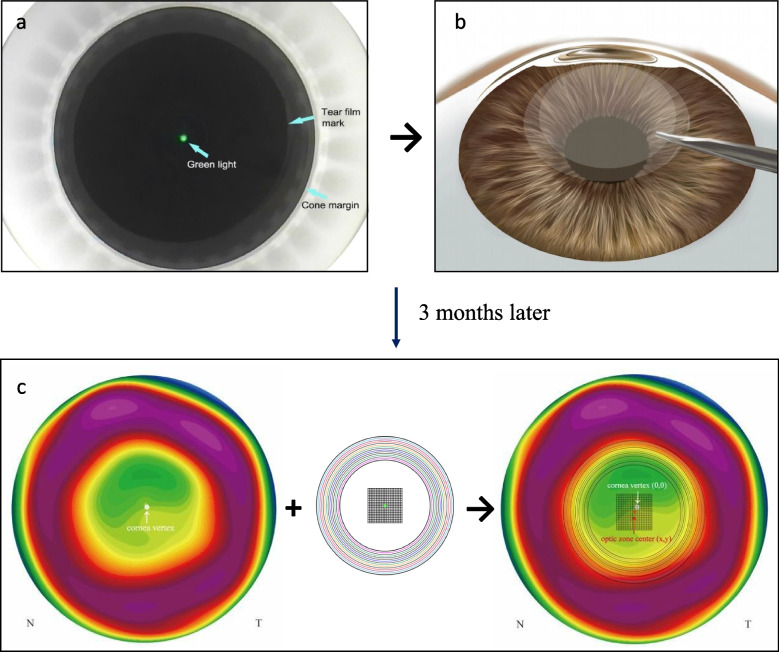
The operation of SMILE surgery (**a**). Tear film mark centration method was used to determine the process of centration. At 80% size, the green light was modified to coincident with the tear film center. green light = treatment center. (**b**). After centration, the operation of femtosecond laser treatment and the lenticule extraction were performed (**c**). Decentration was measured on the tangential curvature difference map, with the corneal vertex shown by the white dot (arrow). A prepared best fitting circle and central grid was superimposed to the tangential curvature difference map to determine the location of the optical zone center (x,y) with reference to the corneal vertex (0,0) by the Adobe. N = nasal; T = temporal

Patients received standardized topical anesthesia via two drops of 0.5% proparacaine hydrochloride (Alcaine; Alcon Laboratories, USA) for the two operated eyes prior to the surgery. The VisuMax femtosecond laser system (Carl Zeiss Meditec AG, Jena, Germany) was employed for all surgeries, set at a power of 500 kHz and a pulse energy of 140 nJ. The decision regarding whether to perform the first surgical procedure on the left or right eye is made randomly before the operation. The surgeon will reposition the patient's head to align both bilateral lateral canthi at the same level, keeping them perpendicular to the body axis, and ensuring that the plane of the iris is parallel to the ground. Before performing laser scanning on each eye, the surgeon will recheck the positioning of the patient's head and eyes. With the patient's eye positioned under a curved suction cone, fixation on a blinking target light, coaxially aligned with the laser beam, was required. The non-operative eye was typically shielded with a foldable surgical sheet. All procedures centralized on the tear film mark center. The patient’s fixation accuracy was enhanced by correlating preoperative reference points of the corneal vertex and pupil center to the intraoperative video (Fig. 1a). Achieving a 60% overlap between the patient’s cornea and suction cone, the surgeon made necessary adjustments. Further refinements ensured the green signal light aligned with the tear film before activating the suction.

The intended superior cap thickness ranged from 110 − 120 mm, with a lenticule diameter of 6.0 − 6.5 mm in both groups. Laser cut energy was set at 140 nJ, with a cap diameter of 7.5 mm and a refractive lenticule diameter of 6.5 mm. A side cut for lenticule access was crafted 90° apart, and the incision located in superior nasal side (140°) with 2 mm width. Following femtosecond laser application, the lenticule was removed through the side cut under microscopy (Fig. 1b). The procedure of femtosecond laser scanning and the lenticule removement is: Laser scanning of the first operated eye, laser scanning of the second operated eye, manual separation of the second operated eye lenticule, and manual separation of the first operated eye lenticule. An experienced surgeon executed all procedures. No complications, such as suction loss or lenticule separation issues, arose. Post-surgery, patients were prescribed levofloxacin 0.5% and loteprednol etabonate 0.5% (Lotemax®) four times daily for two weeks.

### Measurement of decentration

Tangential curvature was procured using the Scheimpflug camera (Pentacam; Oculus Optikgeräte, Wetzlar, Germany) three months postoperatively. Centration accuracy was gauged employing a modified method, as described in prior research [9]. On the tangential topography difference map, the optical zone was delineated up to the mid-peripheral power inflection point. Graphs from this map were imported into Adobe Illustrator software (Adobe Inc., San Jose, CA, USA). A pre-determined best-fitting circle and central grid were overlaid to pinpoint the optical zone center in relation to the corneal vertex. The corneal vertex was set as the (0, 0) point, from which the software generated a coordinate (x, y), denoting the central point of the optical zone. Decentration was examined for horizontal, vertical, and overall shifts. Positive horizontal values denoted nasal shifts, while negative values indicated temporal shifts. For symmetry, x coordinates for left eyes were mirrored on the horizontal axis (Fig. 1c). Preoperative pupillary offset (angle kappa) for each eye was gauged using the Scheimpflug tomography system (Pentacam). An evaluation was also conducted to determine the relationship between vector differences in pupillary offset and variations in decentrations.

### Measurements of wavefront aberrations and MTF

With the Scheimpflug camera (Pentacam), corneal wavefront aberrations were captured. Consistent darkened lighting conditions were maintained for all patient measurements. Each eye underwent three readings per visit, with the optimal acquisition (quality specification = OK) selected for analysis. Coefficients were reviewed for a standardized 6 mm diameter. The root mean square (RMS) value of total HOAs was studied up to the sixth order using an extended set of Zernike polynomials. Specific analyses included spherical aberration (Z4, 0), vertical coma (Z3, -1), and horizontal coma (Z3, 1).

Objective optical quality was ascertained using a ray-tracing aberrometer (iTrace, Tracey Technologies, Houston, TX, USA) both preoperatively and at the three-month review. Modulation transfer function (MTF) values at spatial frequencies of 5, 10, 15, 20, 25, and 30 cycles/degree (c/d) were recorded. Objective MTF measurements were executed with a pupil standardized at 4.0 mm. A seasoned operator performed all measurements.

### Statistical analysis

Data were analyzed using SPSS (Version 20.0; SPSS, Chicago, IL). To determine the significance of differences between the first surgical eyes and subsequent surgical eyes, the Chi-square test was used for categorical variables, and the paired-samples t-test for normally distributed variables. Safety was ascertained by computing a safety index, defined as the CDVA at 3 months divided by the CDVA before surgery. Efficacy was gauged by calculating an efficacy index, determined by the ratio of post-operative UDVA at 3 months to the preoperative CDVA. Linear regression explored the potential association between corneal wavefront aberration, MTF values, and decentered displacement. A *P*-value of less than 0.05 was considered statistically significant.

## Results

### Baseline characteristics

Table 1 summarizes the baseline characteristics of the two groups. The study encompassed 202 subjects (404 eyes), of which 74 were male and 128 were female. The subjects had an average age of 26.4 ± 5.6 years. The ratio of the right versus the left eye to be the first operated eye is 135/67 (66.8%/33.2%). There were no significant disparities in IOP, K1, K2, mean K, CCT, pupil diameter, optical zone, angle kappa, or lenticule thickness between the paired eyes (all *P* > 0.05). Likewise, no substantial differences emerged in the preoperative pupillary offset, inclusive of both the x and y axes, between the eyes operated on.

**Table 1:** Baseline characteristics of the first surgical eyes and the fellow eyes

Characteristic	First eye	Second eye	*P* Value*
Number of eyes	202	202	-
Age,y	26.41 ± 5.47	26.41 ± 5.47	-
Sex, % women	63.4	63.4	-
Intraocular Pressure, mmHg	15.96 ± 1.75	15.80 ± 1.74	0.087
Keratometric power of flat meridian (K1)	42.89 ± 1.30	42.85 ± 1.33	0.079
Keratometric power of steep meridian (K2)	44.10 ± 1.36	44.10 ± 1.35	0.852
Mean curvature power (Mean K)	43.50 ± 1.30	43.48 ± 1.30	0.270
Cornea Central Thickness (CCT), um	542.17 ± 29.11	541.58 ± 28.86	0.370
Pupil Diameter, mm	5.87 ± 0.76	5.83 ± 0.79	0.192
Optical zone, mm	6.47 ± 0.10	6.47 ± 0.13	0.703
Lenticule thickness, μm	118.23 ± 25.55	118.34 ± 26.26	0.898
**Preop pupillary offset, mm**	0.16 ± 0.09	0.16 ± 0.09	0.361
X axis	0.01 ± 0.12	-0.01 ± 0.12	0.249
Y axis	0.05 ± 0.13	0.06 ± 0.12	0.159
**Preop**			
Spherical (D)	-5.24 ± 2.15	-5.14 ± 2.13	0.506
Cylindrical (D)	-0.82 ± 0.60	-0.88 ± 0.80	0.231
Manifest Refraction Spherical Equivalent (MRSE)	-5.64 ± 2.23	-5.52 ± 2.34	0.445
Uncorrected Visual Acuity (UDVA)	1.41 ± 0.34	1.41 ± 0.33	0.884
Corrected Distance Visual Acuity (CDVA)	0.00 ± 0.01	0.00 ± 0.02	0.219
**Postop**			
Spherical (D)	0.07 ± 0.55	0.00 ± 0.56	0.087
Cylindrical (D)	-0.43 ± 0.32	-0.39 ± 0.34	0.129
Manifest Refraction Spherical Equivalent (MRSE)	-0.17 ± 0.57	-0.20 ± 0.60	0.344
Uncorrected Visual Acuity (UDVA)	0.00 ± 0.02	0.00 ± 0.02	0.340
Corrected Distance Visual Acuity (CDVA)	0.00 ± 0.02	0.00 ± 0.01	0.391
Safety index	1.01 ± 0.05	1.01 ± 0.08	0.104
Efficacy index	1.00 ± 0.06	1.01 ± 0.08	0.090

^*^Significance of differences between groups: paired-samples t test

### Safety and efficacy

Both groups exhibited an improvement in uncorrected visual acuity (all *P* < 0.05). There were no significant differences in mean spherical equivalent or mean astigmatism between the groups both pre- and post-operation (all *P* > 0.05). Preoperative manifest refractive spherical equivalent (MRSE) values for the first and second surgical eyes stood at -5.64 ± 2.23 D and -5.52 ± 2.34 D, respectively (*P* = 0.445). Three months post-SMILE surgery, these MRSE values were -0.17 ± 0.57 D for the first surgical eyes and -0.20 ± 0.60 D for the second surgical eyes (*P* = 0.344). The preoperative UDVA (LogMAR) was recorded as 1.41 ± 0.34 for the first surgical eyes and 1.41 ± 0.33 for the second (*P* = 0.884), while postoperative figures stood at 0.002 ± 0.02 for the former and 0.0004 ± 0.02 for the latter (*P* = 0.340). Both pre- and post-operative CDVA (LogMAR) for the two eyes yielded comparable results (all *P* > 0.05). Furthermore, the safety indices for the eyes were 1.01 ± 0.05 and 1.01 ± 0.08 (*P* = 0.104), while efficacy indices registered at 1.00 ± 0.06 and 1.01 ± 0.08 (*P* = 0.090).

### Decentered displacement

**Fig. 2:**
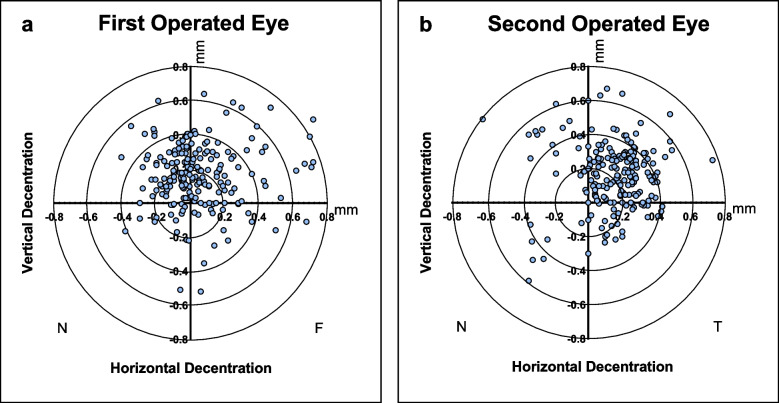
Comparison of the decentered displacement between the first operated eyes (**a**) and the second operated eyes (**b**), in terms of the horizontal decentration, the vertical decentration and total decentration. N = nasal; T = temporal. * Significance of differences between groups: paired-samples t test

Figure 2 presents the total decentered displacement for both the first and second surgical eyes. Displacement values were 0.278 ± 0.17 mm for the first surgical eyes and 0.315 ± 0.15 mm for the second (*P* = 0.002). Horizontal displacements recorded 0.047 ± 0.21 mm for the first surgical eyes and 0.151 ± 0.19 mm for the second (*P* = 0.000), while vertical displacements between the two groups showed no noteworthy differences.

### Wavefront aberrations

**Fig. 3:**
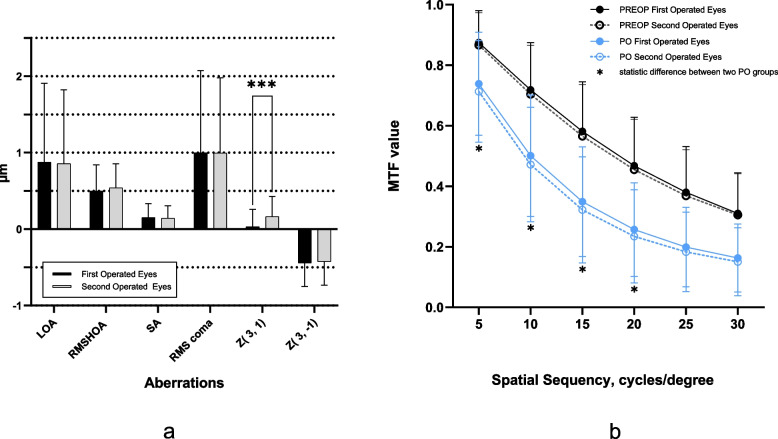
Comparison of induced corneal aberrations (**a**) and modulation transfer function (MTF) values at different spatial sequence (**b**) between the first operated eyes and the second operated eyes. LOA = low-order aberrations; RMS = root mean square; HOAs = high-order aberrations; SA = spherical aberration; Z (3, 1) = horizontal coma; Z (3, -1) = vertical coma; PREOP = pre-operation; PO = post-operation. * Significance of differences between groups: paired-samples t test

Figure 3a illustrates the induced corneal aberrations. Three months post-surgery, distinctions in the induced changes in low aberrations, RMS high-order aberrations, spherical aberration, RMS coma, or vertical coma were not evident between the groups. However, the horizontal coma for the groups were 0.03 ± 0.23 mm and 0.16 ± 0.27 mm, respectively (*P* = 0.000).

### MTF values

Figure 3b depicts the MTF values. Prior to surgery, no discernible differences in MTF values were observed between the groups across all spatial frequencies. Post-surgery, MTF values for the first surgical eyes were relatively higher than those of the second surgical eyes at spatial frequencies of 5, 10, 15, and 20 cycles/degree (c/d) (*P*^5c/d^ = 0.033, *P*^10c/d^ 0.032, *P*^15c/d^ = 0.044, *P*^20c/d^ = 0.049). Notably, as spatial frequency ascended, a gradual decrease in the modulation transfer function (MTF) value emerged between the two groups.

### Linear regression analysis

**Fig. 4:**
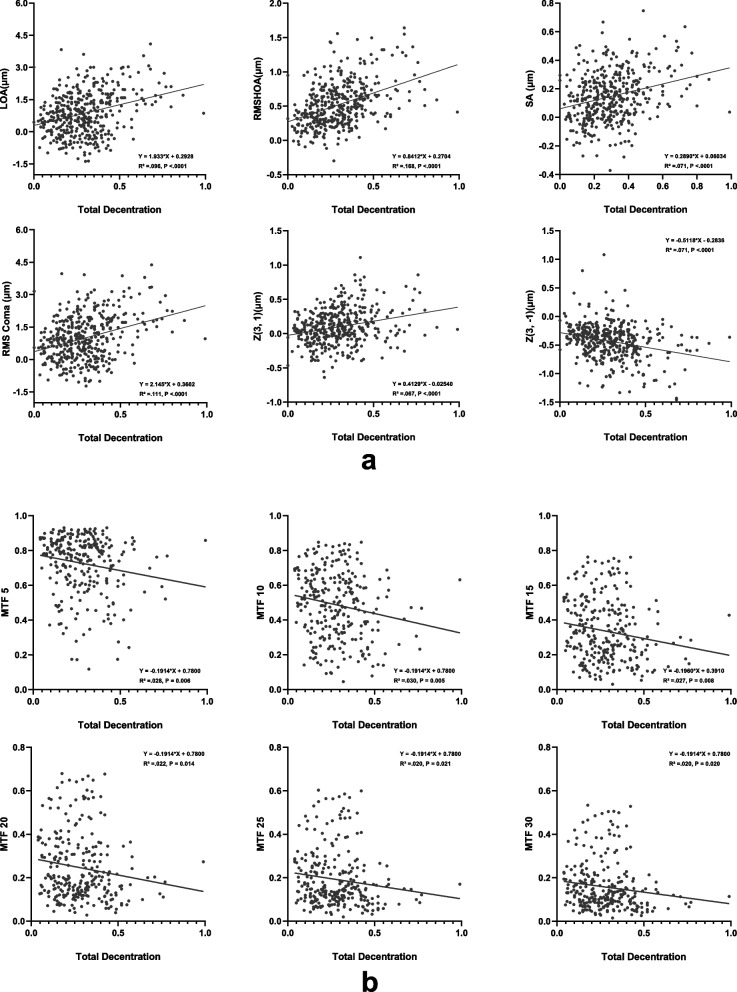
Linear regression analysis between corneal aberrations and decentration (**a**) and linear regression analysis between MTF values and decentration (**b**)

Figure 4a depicts the univariate association between decentered displacement and corneal wavefront aberrations. A clear linear correlation is evident between eccentric displacement and several corneal wavefront aberrations, including RMS high-order aberrations, spherical aberration, RMS coma, vertical coma, and horizontal coma in both groups (all *P* < 0.05). With the escalation of corneal eccentric displacement, there's a concomitant increase in low aberrations, RMS high-order aberrations, spherical aberration, RMS coma, and horizontal coma, and a decrease in vertical coma.

Figure 4b elucidates the linear relationship between MTF value and eccentric displacement. Post-SMILE procedure, the MTF values exhibited a decrease relative to preoperative measures. A discernible trend is observed: as eccentric displacement amplifies, the MTF value diminishes, regardless of changes in spatial sequence.

## Discussion

This study analyzed the postoperative visual quality in patients who underwent consecutive SMILE surgery, comparing the first operated eyes with the second operated ones. While all patients displayed a marked enhancement in postoperative uncorrected visual acuity compared to preoperative metrics, the operation's aftermath was devoid of notable complications. Both eyes presented comparable safety and efficacy outcomes. Intriguingly, the degree of decentration was less pronounced in the first operated eyes than in the second. Moreover, horizontal coma and MTF values at spatial frequencies of 5, 10, 15, and 20 cycles/degree were notably better in the first operated eyes. Linear regression indicates that the differences in horizontal coma and MTF values might be attributed to variations in eccentric displacement.

Consistent with prior research, SMILE surgery has proven effective in enhancing the uncorrected visual acuity in both eyes, consistently demonstrating exceptional safety and efficacy [10, 11]. No discernible differences were observed in visual acuity, safety index, or efficacy index between the two eyes.

Precise centration during laser ablation is pivotal to achieve optimal post-surgical visual outcomes. Decentered ablation might elevate the risk of complications such as postoperative irregular astigmatism, halos, glare, diminished contrast sensitivity, monocular diplopia, and a decline in visual acuity [12]. A multitude of factors could explain why the first operated eyes registered a lower degree of postoperative decentration than the second. The position of the patient's head during surgery may affect the patient's posture on eye movement via Vestibular-ocular reflex (VOR), resulting in the deviation of the patient’s head position during the laser scanning [13, 14]. These movements might vary for each eye operated upon induced by ocular discomfort and stress. Past studies indicate that the first operated eyes experience less ocular discomfort [15]. This could be due to diminished anxiety and heightened awareness of pain during subsequent procedures, making individuals more cognizant of discomfort [16, 17]. However, certain literature about corneal refractive surgery has also documented no discernible disparity in photorefractive keratectomy( PRK) in terms of patients' cooperation and perceived pain during first and second eye [14]. No statistical differences in pain scores were observed for the postoperative period after laser in situ keratomileusis [18]. One possible guess: Once the first eye surgery completed, patients could expect similar series of process during the second eye surgery. This anticipation can potentially lead to increased anxiety and a decreased ability to tolerate pain.

Additional to the physiological factors, the order of surgical operations might influence short-term microdistortions of the Bowman's layer post-SMILE surgery, potentially impacting short-term results [15]. We also speculate the anesthesia factors involved. In order to reduce the impact of repeated eyelid opening on patient's psychological tension, we uniformly administered bilateral proparacaine eye drops for ocular anesthesia. The total duration of bilateral eye surgery is around 10 min, which within the effective time range of proparacaine eye drops [19]. However, the effectiveness may gradually decrease over time. Therefore, patients may experience more pronounced pain during the second eye surgery, leading to an increased decentered displacement. Although significant disparities in horizontal and overall eccentricity were observed between the groups, vertical eccentricity remained consistent. This observation could be linked to the higher prevalence of horizontal microsaccades during eye fixation [20].

The focus in refractive surgery has evolved from primarily prioritizing the procedure's safety and efficacy to enhancing the patients' visual quality. Evaluating advanced corneal visual quality typically employs wavefront aberrations, with particular attention to high-order aberrations (HOAs), while visual outcomes for eyes are generally represented by MTF values. Consistent with prior research, aberrations induced by decentration are primarily HOAs rather than lower-order aberrations such as astigmatism [21]. This study's findings align with this observation, noting minimal variance in lower-order aberrations, with the main difference in HOAs manifesting in horizontal coma.

MTF values denote the finest human eye resolution across varied spatial frequencies. Higher MTF values suggest superior optical quality [22]. In this analysis, the MTF values in the first surgical eyes at spatial frequencies of 5, 10, 15, and 20 cycles/degree surpassed those of the second surgical eyes postoperatively. Though LASIK surgeries can witness postoperative increases in low-order MTF due to factors like halo or interface refraction, the incidence in SMILE surgeries is reportedly minimal [23].

Past studies have highlighted a robust correlation between decentration and the alterations in wavefront aberration and contrast sensitivity post-SMILE surgery [24]. From this research, it can be inferred that HOAs and reduced MTF values may be associated with a significant rise in total eccentric displacement. While minor intraoperative decentered displacements after SMILE might not considerably influence postoperative visual acuity and refractive outcomes, they could diminish the effectiveness of corrected HOAs and even introduce corneal HOAs [25]. Emphasis on the relationship between horizontal decentration and resulting horizontal coma has been accentuated by multivariate correlation analyses [23], and the vertical coma prominence has also been reported [26]. The correlation between increased decentration and decreased vertical coma may be explained by no statistically difference shown in vertical decenter displacement between two eyes. High MTF values, often indicative of enhanced contrast sensitivity, are typically associated with optimal centration. Fluctuations in these values could be attributed to eccentric displacement, which signifies a deviation from the envisioned visual center, with more severe deviations correlating with reduced visual quality [27].

In clinical settings, it may be beneficial to prioritize the eye with a significant influence on overall visual quality as the first surgical target, such as the eye with superior visual acuity or the dominant one. For professionals like surgeons and athletes, where precise visual performance is paramount, determining the ideal eye for initial surgery is imperative.

This study encompasses data from 202 samples, all treated by a singular experienced physician. Multiple assessments of visual parameters, both pre- and post-surgery (2–3 repetitions), were conducted to ensure consistent, reliable results, highlighting the measures' reliability and repeatability.

However, this study is not without limitations. Despite rigorous quality control efforts to negate surgical bias, patient-specific factors like palpebral aperture size, anxiety levels, Bell's reflex strength, and corneal sensitivity might introduce unintended biases. Moreover, incorporating subjective questionnaires to gauge pain perception and other emotions during surgeries could offer deeper insights. Patient feedback post-operation might also furnish supportive information. It's also worth noting that this study's data scope extends only to three months post-operation. Given that MTF values and corneal aberrations might revert to their preoperative state six months post-surgery or later, investigations with extended postoperative timelines are essential.

## Conclusions

In summary, the study underscores the increasing focus on enhancing advanced visual quality in patients by evaluating high-order aberrations (HOAs) and MTF values. The order of surgical interventions appears to influence post-SMILE visual outcomes, especially concerning parameters like eccentric displacement, which subsequently impacts corneal vertical coma and MTF values. It was observed that the first surgical eyes manifested superior MTF values post-operatively compared to the second. A distinct correlation was also evident between alterations in wavefront aberrations and decentration post-SMILE surgery. These findings accentuate the importance of extended post-operative evaluations and the customization of surgical approaches based on individual patient characteristics.

## Data Availability

The datasets used or analysed during the current study are available from the corresponding author on reasonable request.

## Abbreviations

SMILE: Small-incision lenticule extraction
MTF: Modulation transfer function
HOA: High-order aberrations
RMS: Root mean square
LASIK: Laser in situ keratomileusis
MRSE: Manifest Refraction Spherical Equivalent
UDVA: Uncorrected distance visual acuity
CDVA: Corrected distance visual acuity
CCT: Central corneal thickness
